# Case of Catalepsy Combined with Mania

**Published:** 1828-10

**Authors:** Jos. Canham


### CATALEPSY.

*Case of Catalepsy combined with Mania.* By Jos. Canham, M.D.

In one of the late fasciculi [fasc. iv. of No. 18,] of the Medico-Chirurgical Review, I find detailed a curious case of catalepsy connected with mania. The only case of the disease I ever saw was likewise combined with mania. I am sorry that, from not being able to find the notes I took of the case, I cannot give you as minute an account of it as I wish.

B. M., aged about twenty, (apprentice to his father, a bricklayer,) became deranged, and in a few days was sent to St. Luke's, where he remained a year, and was then discharged, and placed by his friends in Warburton's asylum. In a few weeks his friends took him home. Two days after his return to Stevenage (where I then resided), he was, during dinner, suddenly attacked with catalepsy. When I saw him, which was almost immediately, he sat perfectly motionless; his eyes open and immovable; vision apparently lost, as he would allow a finger to be placed upon the eye without closing the eyelids; insensible to sound; face and hands quite pale, having the appearance of white wax; temperature much reduced; pulse at the wrist not to be felt. Upon an attempt being made to abstract blood, to the surprise of every one present, the arm, upon being raised, retained that position after the hand which had supported it was removed. One leg was now placed at right angles with the trunk: it would not retain this position, but, upon being placed about a foot from the ground, remained there, the patient being seated upon a chair of the usual height. He was now carried to bed, having every appearance of a dead man. I could not see that he breathed, but, upon applying the hand to the chest, a slight motion of the ribs was perceptible.

A very small quantity of blood was procured from the arm, not more than an ounce, (he did not appear to feel the incision with the lancet.) A few drops of ether, with camphorated mixture, was given him, but he made no effort to swallow. Flannels were heated and applied, and friction used, without producing any effect.

I saw him every two hours from the time I first visited him, which was about two P.M. Until twelve, he remained without any change. He was likewise seen by Mr. JONES, who was then practising at Stevenage.

Early the following morning, he was in the same state, and had remained so during the night, according to the account of his brother and those who sat up with him. At about nine o'clock, a slight perspiration appeared on the face, which increased to a most profuse sweat over the whole body, and he shortly afterwards turned in bed, and soon spoke.—The excretions of urine, &c. were suppressed during the continuance of the cataleptic symptoms.

He now became morose and melancholy, which was the state he was in prior to this attack. I saw him for some days afterwards, and he continued in this state.

About six months afterwards, I was sent for to see him: he had then, without any interference of art, regained his intellect, but was lame from the nails of the great toes having penetrated the skin on each side. When these were cured by the application of caustic, he returned to work.

It is about three years since the attack of catalepsy, and he is now perfectly well, as I heard of him very lately. His father was several times insane.

*Ramsgate; September 14th, 1828.*

